# The impact of high-altitude migration on cardiac structure and function: a 1-year prospective study

**DOI:** 10.3389/fphys.2024.1459031

**Published:** 2024-08-30

**Authors:** Ming-Dan Deng, Xin-Jie Zhang, Qin Feng, Rui Wang, Fen He, Feng-Wu Yang, Xian-Mei Liu, Fei-Fei Sun, Jie Tao, Shuang Li, Zhong Chen

**Affiliations:** ^1^ Department of Ultrasound, The General Hospital of Western Theater Command, Chengdu, Sichuan, China; ^2^ Department of Cardiology, The General Hospital of Western Theater Command, Chengdu, Sichuan, China

**Keywords:** high altitude, hypoxia, echocardiography, cardiac remodeling, cardiac function

## Abstract

**Introduction:**

The trend of human migration to terrestrial high altitudes (HA) has been increasing over the years. However, no published prospective studies exist with follow-up periods exceeding 1 month to investigate the cardiac change. This prospective study aimed to investigate the changes in cardiac structure and function in healthy young male lowlanders following long-term migration to HA.

**Methods:**

A total of 122 Chinese healthy young males were divided into 2 groups: those migrating to altitudes between 3600 m and 4000 m (low HA group, n = 65) and those migrating to altitudes between 4000 m and 4700 m (high HA group, n = 57). Traditional echocardiographic parameters were measured at sea level, 1 month and 1 year after migration to HA.

**Results:**

All 4 cardiac chamber dimensions, areas, and volumes decreased after both 1 month and 1 year of HA exposure. This reduction was more pronounced in the high HA group than in the low HA group. Bi-ventricular diastolic function decreased after 1 month of HA exposure, while systolic function decreased after 1 year. Notably, these functional changes were not significantly influenced by altitude differences. Dilation of the pulmonary artery and a progressive increase in pulmonary artery systolic pressure were observed with both increasing exposure time and altitude. Additionally, a decreased diameter of the inferior vena cava and reduced bicuspid and tricuspid blood flow velocity indicated reduced blood flow following migration to the HA.

**Discussion:**

1 year of migration to HA is associated with decreased blood volume and enhanced hypoxic pulmonary vasoconstriction. These factors contribute to reduced cardiac chamber size and slight declines in bi-ventricular function.

## 1 Introduction

Transportation network advancements and improved medical care have facilitated an upsurge in human presence at terrestrial high altitudes (HA) (Luks and Hackett, 2022). These regions are characterized by distinct features, including low temperatures, low humidity, high ultraviolet radiation exposure, and most notably, hypobaric hypoxia. A lack of oxygen significantly impacts cellular function. Notably, the cardiovascular system, which is responsible for oxygen transport, plays a crucial role in enabling human adaptation to the challenging HA environments, as evidenced by research findings (Naeije, 2010; Richalet et al., 2024).

Changes in cardiac structure and function are an integral part of the cardiovascular system’s response to the challenges of high-altitude environments (Williams et al., 2022). These challenges include activation of the sympathetic nervous system, hypoxic pulmonary vasoconstriction, and myocardial oxygen deprivation, all of which contribute to increased afterload on the right ventricle (RV) and impaired left ventricular (LV) filling (Vogel and Harris, 1967). Acute responses in the first week at HA are well-documented, with reported increases in heart rate (HR) and decreases in stroke volume (SV) (Maufrais et al., 2017; Boussuges et al., 2000; Holloway et al., 2011). While LV diastolic function declines, LV systolic function remains largely preserved under acute hypoxia. However, changes in RV structural and functional parameters remain debatable (Maufrais et al., 2017; Yuan et al., 2021). Lifelong adaptation to HA has also been extensively studied. Long-term hypoxic exposure leads to remodeling of the pulmonary artery and RV, resulting in sustained pulmonary hypertension that cannot be readily reversed (Penaloza and Arias-Stella, 2007; Hultgren and Miller, 1967). Additionally, chronic mountain sickness patients, characterized by excessive erythropoiesis and debilitating symptoms, reportedly exhibit an adaptive decrease in RV systolic function (Pratali et al., 2013).

Individuals who consider migrating to high-altitude plateaus are concerned about the long-term progression of cardiac changes at different altitudes. This information can aid them in planning their itineraries safely and effectively. However, limited cross-sectional studies provide the only existing data, and several cardiac parameters in these studies exhibit inconsistencies (Liu et al., 2022; Chen et al., 2022). To date, no published prospective studies exist with follow-up periods exceeding 1 month, attributed to the logistical challenges associated with tracking subjects over extended periods in the harsh HA environments. We hypothesized that the LV and RV function would be preserved after 1 month of migration to HA but impaired after 1 year of migration to HA. Therefore, the present study aimed to conduct a prospective investigation of cardiac changes in healthy male lowlanders after 1 month and 1 year of migration to HA at different altitudes.

## 2 Materials and methods

### 2.1 Study participants

**FIGURE 1:**
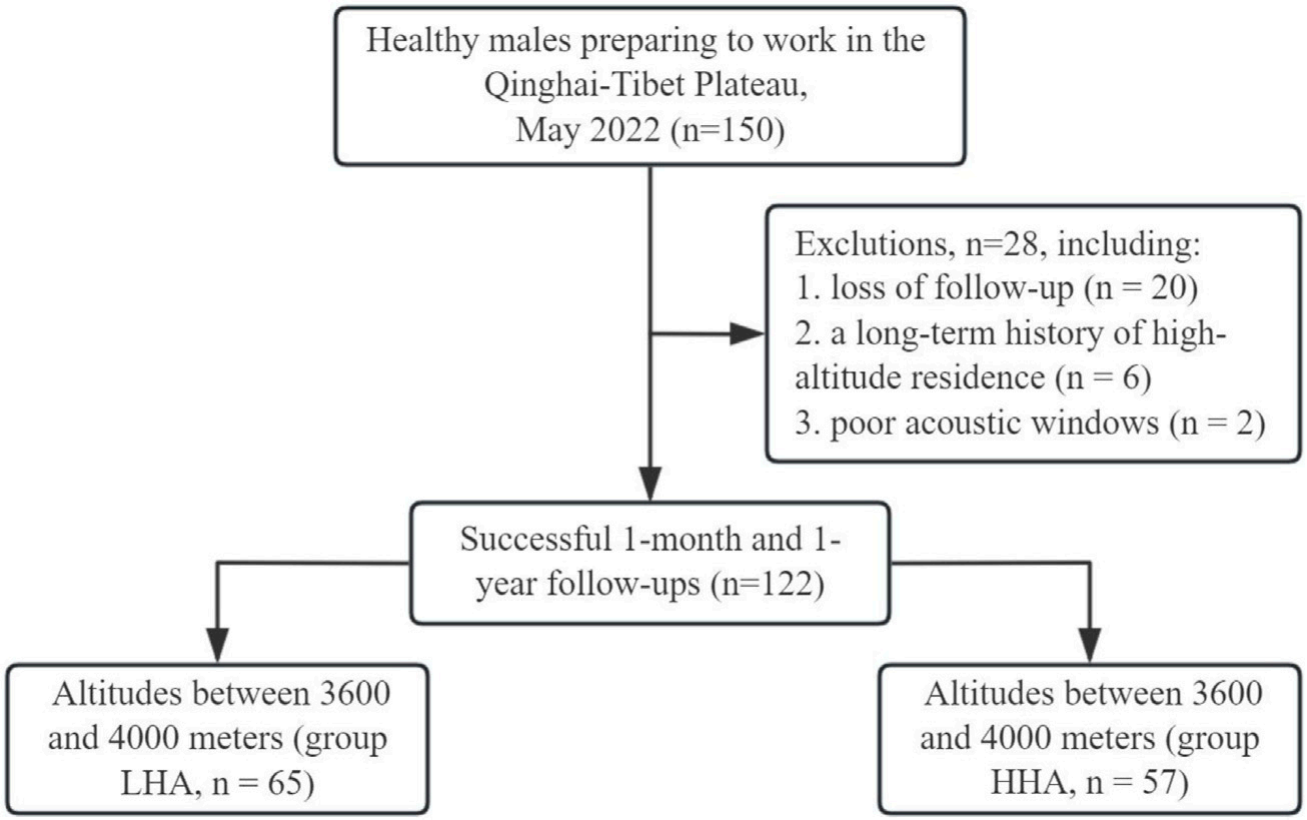
Flowchart of the study of the enrolled participants.

Lowlanders who were preparing to work on the Qinghai‒Tibet Plateau (altitude >3,600 m) for a long time were consecutively recruited in Chongqing (altitude <500 m) in May 2022. The study population consisted of civilians who were engaged in construction. They underwent a comprehensive medical examination before recruitment, and those with conditions such as patent foramen ovale or other heart diseases detected by conventional echocardiography have been excluded from working at high altitudes. The inclusion criteria were as follows: (Luks and Hackett, 2022): Adult males ages 18–65 years, and (Naeije, 2010) born in a low-altitude place (altitude <1,000 m), with (Richalet et al., 2024) no history of cardiovascular disease or (Williams et al., 2022) chronic conditions affecting heart function. The exclusion criteria included (Luks and Hackett, 2022) a long-term history of high-altitude residence; (Naeije, 2010); poor acoustic windows; (Richalet et al., 2024); loss to follow-up. The recruitment pathway is shown in Figure 1. Ultimately, 122 males with successful 1-month and 1-year follow-ups were prospectively enrolled in this study. Following an initial acclimatization period of several days at high altitude, they engaged in a standardized low-intensity and mid-intensity routine infrastructure work regimen for 6 h daily, 5 days per week. Notably, the participants resided continuously at their high-altitude workplaces for a full year without returning to lower elevations. The study protocol was approved by the Ethics Committee of The General Hospital of Western Theater Command of PLA (approval number: 2022EC3-ky062) and was performed according to the Declaration of Helsinki. Written informed consent was obtained from all participants.

### 2.2 Research design

All participants undertook a 30-h train journey to the Qinghai‒Tibet Plateau and then dispersed to work at different altitudes. To investigate the influence of altitude, participants were divided into 2 groups: the low-altitude group (LHA, n = 65), comprising individuals residing at altitudes between 3,600 m and 4,000 m, and the high-altitude group (HHA, n = 57), comprising individuals residing at altitudes between 4,000 m and 4,700 m. Baseline measurements were conducted in Chongqing 2 weeks before departure. Subsequent investigations were conducted in Tibet at 1 month (HA-1M) and 1 year (HA-1Y) after arrival at the corresponding altitudes. The data collection duration for each phase was controlled within 1 week.

### 2.3 Clinical measurements

Systolic blood pressure, diastolic blood pressure, pulsed oxygen saturation, and HR were measured after a minimum of 5 min of rest using a sphygmomanometer (HEM-6200, OMRON) and a pulse oximeter (PO2, Lepu Medical), respectively.

### 2.4 Echocardiography

After at least 10 min of rest, transthoracic echocardiograms were acquired in the left lateral decubitus position using portable ultrasound machines (M9 and M10; Mindray) equipped with low-frequency (1–5 MHz) phased array transducers by 2 well-trained ultrasound doctors.

Cardiac dimensions and functions were evaluated following the American Society of Echocardiography standards (Lang et al., 2015; Rudski et al., 2010). Detailed protocols of echocardiographic measurements are present in Supplementary Material. The LV ejection fraction was manually measured using Simpson’s biplane method. Cardiac output was calculated by multiplying SV by HR. LV mass was calculated using the linear method, and the LV mass index was determined by dividing LV mass by the body surface area. The left atrial volume was calculated using the biplane method, and the left atrial volume index was calculated by dividing the left atrial volume by the body surface area. Fraction area change and tricuspid annular plane systolic excursion (TAPSE) were measured to assess RV systolic function. The peak velocities of the mitral and tricuspid early (E) and late (A) waves were measured, and the E/A ratio was calculated to assess LV and RV diastolic function. The right atrial (RA) pressure was estimated based on the diameter of the inferior vena cava (IVC) and its inspiratory variation. The tricuspid regurgitation pressure gradient (TRPG) was calculated from the velocity of tricuspid regurgitation (TRV) using the simplified Bernoulli equation. Pulmonary artery systolic pressure (PASP) was estimated by adding TRPG to the RA pressure.

### 2.5 Statistical analysis

Statistical analyses were performed using SPSS 22.0 software (SPSS Inc., Chicago, IL), with significance set at *p* < 0.05. Descriptive statistics for categorical variables included frequencies and percentages, and for continuous variables, means ± SD. The normality of continuous variables was assessed using the Kolmogorov-Smirnov test.

Baseline group comparisons employed chi-square tests for categorical data and independent t-tests for continuous data, given compliance with normal distribution. Longitudinal differences in clinical and echocardiographic measures across time points (baseline, 1-month, and 1-year) and between altitude groups (low-altitude vs high-altitude) were analyzed using 2-way repeated measures ANOVA, focusing on interaction and main effects.

Significant findings from the ANOVA were further explored using *post hoc* Bonferroni correction to adjust for multiple comparisons if a significant main effect was detected with F > 3.86 and *p* < 0.05. Results were reported as exact *p*-values, and 95% confidence intervals (CIs) were provided where relevant.

By incorporating 95% CIs, this approach ensures a comprehensive and transparent analysis of the statistical effects of high-altitude migration on cardiac functions over time while maintaining rigorous statistical standards.

## 3 Results

### 3.1 Clinical characteristics

A total of 122 males were prospectively enrolled in this study, of whom 65 were in the LHA group and 57 were in the HHA group. The mean altitudes of the LHA and HHA groups were 3,714.8 ± 122.7 m and 4,290.8 ± 175.7 m, respectively. The locations and altitudes are detailed in Table 1. No significant differences were found between the 2 groups in terms of age, height, body mass index, body surface area, or smoking history (Table 2).

**TABLE 1:** Location and altitude.

Group	Location	Altitude (m)	Number of participants	Total
Low HA	Lhasa city	3,650	49 (40.2%)	65 (53.3%)
	Lhasa city	3,890	16 (13.1%)	
High HA	Shigatse city	4,118	17 (13.9%)	57 (46.7%)
	Shigatse city	4,260	15 (12.3%)	
	Shigatse city	4,267	5 (4.1%)	
	Shigatse city	4,306	10 (8.2%)	
	Shigatse city	4,502	3 (2.4%)	
	Shigatse city	4,667	4 (3.3%)	
	Shigatse city	4,700	3 (2.4%)	

Data are expressed as number (percentage).

**TABLE 2:** Baseline clinical characteristics.

	Total (n = 122)	LHA (n = 65)	HHA (n = 57)	*p*-value
Age (year)	21.5 ± 1.5	21.4 ± 1.4	21.5 ± 1.6	0.113
Height (cm)	172.4 ± 5.5	173.0 ± 5.7	171.8 ± 5.3	0.828
Weight (kg)	63.9 ± 7.4	64.3 ± 7.2	63.5 ± 7.5	0.759
BMI (kg/m^2^)	21.5 ± 2.1	21.4 ± 1.9	21.5 ± 2.4	0.067
BSA (m2)	1.7 ± 0.1	1.7 ± 0.1	1.7 ± 0.1	0.681
Smoking	72 (59%)	39 (60%)	33 (57%)	0.814
Altitude (m)	3,983.9 ± 324.8	3,714.8 ± 122.7	4,290.8 ± 175.7	NA

Data are expressed as mean ± SD, or as number (percentage).

BMI, body mass index; BSA, body surface area.

*p* value, group LHA, versus HHA.

NA, not assessed.

Changes in clinical characteristics are shown in Table 3. In HA-1M, the SpO₂ dropped significantly in the LHA group and further decreased in the HHA group. This decrease was maintained in HA-1Y. HR and blood pressure increased significantly in both groups in HA-1M, with no further significant increase in HA-1Y. However, diastolic blood pressure decreased in the HHA group in HA-1Y compared to that in HA-1M. In HA-1M, the HR was higher in the HHA group compared to the LHA group.

**TABLE 3:** Clinical characteristics at sea level and after 1 month and 1 year of migration to HA.

	SL		HA-1M		HA-1y		ANOVA *p*-value		
	LHA	HHA	LHA	HHA	LHA	HHA	Time	Group	Interaction
SpO_2_ (%) (95% CI)	97.7 ± 0.6 (97.6–97.9)	97.8 ± 0.6 (97.6–98.0)	91.8 ± 2.7* (91.1–92.5)	89.5 ± 3.2*‡ (88.7–90.2)	92.2 ± 3.1* (91.5–93.0)	89.3 ± 2.9*‡ (88.5–9*0.1)	<0.001	<0.001	<0.001
HR (beats/min) (95% CI)	61.7 ± 8.0 (59.7–63.7)	63.2 ± 8.1 (60.0–64.3)	69.3 ± 12.9* (66.1–72.6)	74.8 ± 13.6*‡ (71.3–78.3)	71.2 ± 10.4* (68.6–73.7)	7*4.1 ± 10.4* (71.4–76.9)	<0.001	0.041	0.096
SBP (mmHg) (95% CI)	112.2 ± 10.1 (109.8–114.6)	113.1 ± 9.1 (110.5–115.6)	117.3 ± 7.1* (115.4–119.3)	117.6 ± 8.7* (115.6–119.7)	118.1 ± 8.3* (115.7–120.4)	117.8 ± 10.6* (115.3–120.2)	<0.001	0.802	0.846
DBP (mmHg) (95% CI)	61.0 ± 7.1 (59.3–62.8)	62.1 ± 7.0 (60.3–63.9)	70.7 ± 9.2* (68.4–72.9)	73.4 ± 8.8* (71.0–75.8)	70.7 ± 8.3* (68.7–72.7)	69.4 ± 7.9*† (67.3–71.6)	<0.001	0.448	0.055

Data are expressed as mean ± SD.

CI, confidence interval; SpO2, pulse oxygen saturation; HR, heart rate; SBP, systolic blood pressure; DBP, diastolic blood pressure.

*p < 0.05 vs. SL; †*p* < 0.05 vs. 1 month; ‡*p* < 0.05 LHA, vs. HHA.

### 3.2 Left heart echocardiographic parameters

**FIGURE 2:**
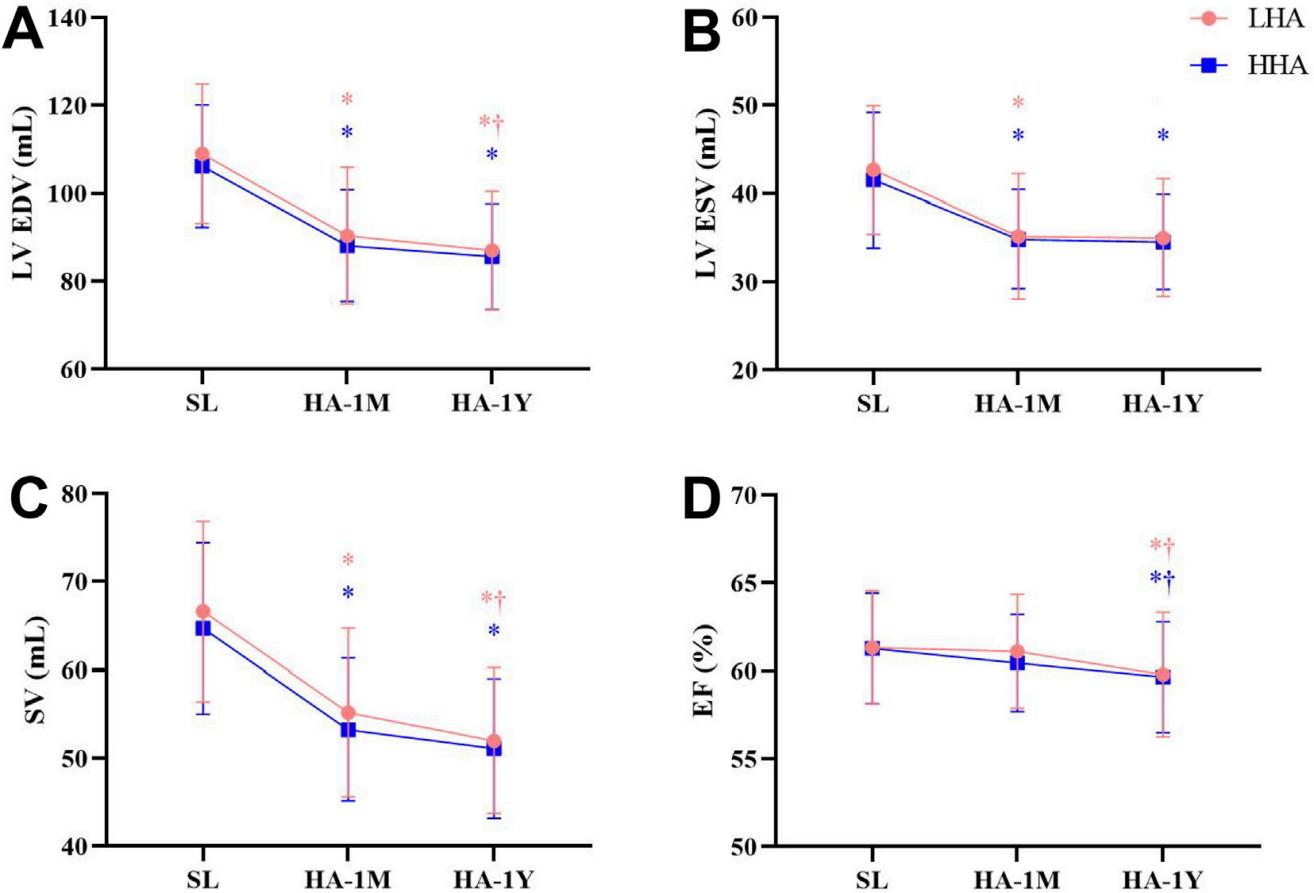
Echocardiographic parameters of the left heart at sea level and after 1 month and 1 year of migration to HA: **(A)** Left ventricular end-diastolic volume (LV EDV). **(B)** Left ventricular end-systolic volume (LV ESV). **(C)** Stroke volume (SV). **(D)** Ejection fraction (EF) *p < 0.05 vs. SL; †*p* < 0.05 vs. 1 month.

The left heart echocardiographic parameters are presented in Table 4; Figure 2, no significant difference was observed between the LHA and HHA groups at any time point. The end-diastolic dimension and volume of the LV, the LA volume, and the SV significantly decreased in HA-1M in both groups. In the LHA group, there was a further decrease in these parameters in HA-1Y, while the HHA group showed no further change. Both groups experienced a significant decrease in ejection fraction in HA-1Y, with no change in HA-1M. Notably, cardiac output decreased only in the LHA group during HA-1Y. The LA dimension and volume indices, LV end-systolic volume, LV mass, LV mass index, bicuspid E velocity, bicuspid A velocity, and bicuspid E/A ratio all decreased in HA-1M but remained unchanged in HA-1Y in both groups. Both interventricular septal thickness and LV posterior wall thickness remained unchanged throughout the year in both groups.

**TABLE 4:** Echocardiographic parameters of the left heart at sea level and after 1 month and 1 year of migration to HA.

	SL		HA-1M		HA-1y		ANOVA *p*-value		
	LHA	HHA	LHA	HHA	LHA	HHA	Time	Group	Interaction
LA APD (mm) (95% CI)	31.6 ± 2.7 (31.0–32.2)	31.0 ± 2.4 (30.3–31.6)	28.1 ± 2.2* (27.7–28.6)	27.6 ± 2.2* (27.0–28.2)	28.5 ± 2.4* (27.8–29.1)	28.2 ± 2.5* (27.5–28.9)	<0.001	0.221	0.717
LAV (mL) (95% CI)	40.2 ± 7.4 (38.5–42.0)	39.1 ± 7.0 (37.2–41.0)	32.9 ± 6.3* (31.6–34.1)	31.2 ± 3.7* (29.9–32.6)	31.7 ± 5.8*† (30.4–32.9)	30.2 ± 4.0* (28.9–31.6)	<0.001	0.108	0.840
LAVi (mL/m2) (95% CI)	23.3 ± 3.9 (22.4–24.3)	22.9 ± 4.0 (21.9–24.0)	19.0 ± 3.3* (18.3–19.7)	18.4 ± 2.3* (17.7–19.2)	18.4 ± 3.1* (17.7–19.0)	17.8 ± 2.4* (17.0–18.5)	<0.001	0.275	0.897
IVST (mm) (95% CI)	8.5 ± 0.7 (8.4–8.7)	8.5 ± 0.8 (8.3–8.7)	8.6 ± 0.8 (8.3–8.8)	8.5 ± 1.0 (8.3–8.7)	8.5 ± 0.8 (8.3–8.7)	8.5 ± 0.9 (8.3–8.7)	0.960	0.795	0.833
LV PWT (mm) (95% CI)	8.1 ± 0.6 (8.0–8.3)	8.0 ± 0.8 (7.8–8.1)	8.0 ± 0.7 (7.8–8.2)	7.8 ± 0.7 (7.7–8.0)	8.0 ± 0.9 (7.7–8.2)	8.1 ± 0.7 (7.9–8.3)	0.331	0.520	0.133
LV EDD (mm) (95% CI)	48.0 ± 3.3 (47.3–48.8)	47.7 ± 2.8 (46.9–48.5)	44.8 ± 3.0* (44.1–45.5)	43.9 ± 2.7* (43.2–44.7)	43.9 ± 3.0*† (43.3–44.6)	43.5 ± 2.7* (42.8–44.2)	<0.001	0.247	0.288
LV EDV (mL) (95% CI)	109.0 ± 15.9 (105.3–112.7)	106.2 ± 14.0 (102.3–110.1)	90.3 ± 15.6* (86.8–93.9)	88.1 ± 12.7* (84.3–91.9)	87.0 ± 13.5*† (83.8–90.1)	85.6 ± 12.0* (82.2–89.0)	<0.001	0.342	0.787
LV ESV (mL) (95% CI)	42.7 ± 7.3 (40.8–44.5)	41.5 ± 7.7 (39.6–43.5)	35.2 ± 7.1* (33.6–36.7)	34.8 ± 5.6* (33.1–36.5)	35.0 ± 6.6* (33.5–36.5)	34.5 ± 5.4* (32.9–36.1)	<0.001	0.532	0.701
SV (mL/beat) (95% CI)	66.6 ± 10.2 (64.2–69.1)	64.7 ± 9.7 (62.1–67.3)	55.2 ± 9.6* (53.0–57.4)	53.3 ± 8.1* (50.9–55.6)	52.0 ± 8.3*† (50.0–54.0)	51.1 ± 7.9* (49.0–53.2)	<0.001	0.260	0.707
EF (%) (95% CI)	61.3 ± 3.2 (60.6–62.1)	61.3 ± 3.1 (60.5–62.1)	61.1 ± 3.2 (60.4–61.9)	60.5 ± 2.8 (59.7–61.2)	59.8 ± 3.6*† (59.0–60.6)	59.6 ± 3.2*† (58.8–60.5)	<0.001	0.511	0.586
CO (L/min) (95% CI)	4.1 ± 0.8 (3.9–4.3)	4.0 ± 0.8 (3.8–4.2)	3.8 ± 0.9 (3.6–4.0)	3.9 ± 0.8 (3.7–4.2)	3.7 ± 0.7* (3.5–3.9)	3.80.7 (3.6–4.0)	<0.001	0.638	0.370
LVM (g) (95% CI)	134.2 ± 17.5 (129.6–138.8)	130.9 ± 20.0 (126.0–135.8)	118.9 ± 19.3* (114.3–123.6)	113.0 ± 18.6* (108.0–118.0)	114.1 ± 20.4* (109.3–118.9)	113.5 ± 18.3* (108.4–118.6)	<0.001	0.261	0.248
LVMi (g/m2) (95% CI)	77.9 ± 9.2 (75.3–80.4)	76.8 ± 11.4 (74.1–79.4)	68.6 ± 10.0* (66.2–71.0)	66.4 ± 9.5* (63.8–68.9)	66.0 ± 10.3* (63.6–68.5)	66.4 ± 9.2* (63.8–69.0)	<0.001	0.488	0.392
Bicuspid E (cm/s) (95% CI)	88.3 ± 18.3 (84.5–92.1)	88.1 ± 12.9 (84.0–92.1)	66.8 ± 12.8* (63.8–69.8)	64.8 ± 11.5* (61.6–68.0)	66.8 ± 13.1* (63.6–70.0)	66.6 ± 13.4* (63.2–70.1)	<0.001	0.693	0.711
Bicuspid A (cm/s) (95% CI)	46.7 ± 9.4 (44.4–49.1)	46.2 ± 9.9 (43.7–48.7)	41.8 ± 9.7* (39.4–44.2)	41.7 ± 9.8* (39.2–44.3)	39.3 ± 7.7* (37.3–41.3)	40.6 ± 8.8* (38.5–42.8)	<0.001	0.844	0.661
Bicuspid E/A ratio (95% CI)	2.0 ± 0.5 (1.8–2.1)	2.0 ± 0.4 (1.8–2.1)	1.6 ± 0.4* (1.5–1.7)	1.6±0.4* (1.5–1.7)	1.7±0.4* (1.6–1.8)	1.7±0.4* (1.6–1.8)	<0.001	0.868	0.785

Data are expressed as mean ± SD.

CI, confidence interval; LA APD, left atrial anteroposterior diameter; LAV, left atrial volume; LAVi, left atrial volume index; IVST, interventricular septal thickness; LV PWT, left ventricular posterior wall thickness; LV EDD, left ventricular end-diastolic diameter; LV EDV, left ventricular end-diastolic volume; LV ESV, left ventricular end-systolic volume; SV, stroke volume; EF, eject fraction; CO, cardiac output; LVM, left ventricular mass; LVMi, left ventricular mass index.

*p < 0.05 vs. SL; †*p* < 0.05 vs. 1 month; ‡*p* < 0.05 LHA, vs. HHA.

### 3.3 Right heart echocardiographic parameters

**FIGURE 3:**
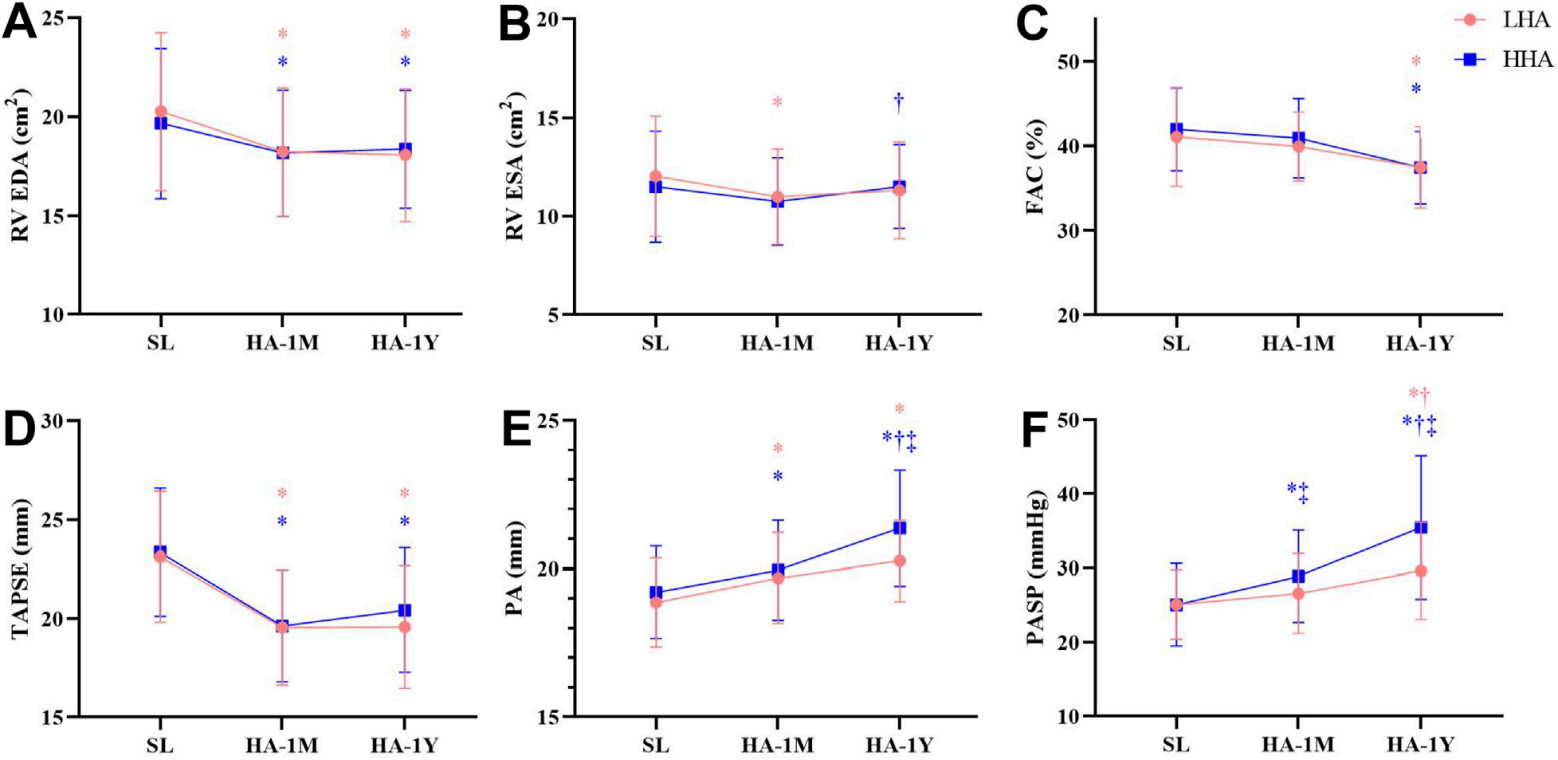
Echocardiographic parameters of the right heart at sea level and after 1 month and 1 year of migration to HA. **(A)** Right ventricular end-diastolic area (RV EDA). **(B)** Right ventricular end-systolic area (RV ESA). **(C)** Fraction area change (FAC). **(D)** Tricuspid annular plane systolic excursion (TAPSE). **(E)** Pulmonary artery (PA). **(F)** Pulmonary artery systolic pressure (PASP). *P < 0.05 vs. SL; †*P* < 0.05 vs. 1 month; ‡*P* < 0.05 LHA vs. HHA.

The right heart echocardiographic parameters are presented in Table 5; Figure 3. In both groups, the RA dimension and area significantly decreased in HA-1M and further decreased in HA-1Y for the LHA group, but not the HHA group. The mid-cavity and outflow tract dimension of the right ventricle, RV end-diastolic area, and tricuspid E/A ratio decreased in HA-1M and remained unchanged in both groups at HA-1Y. The RV free wall thickness remained unchanged across both groups and time points. While fractional area change decreased in HA-1Y, TAPSE only decreased in HA-1M, remaining unchanged in both groups at HA-1Y. Both groups experienced a decrease in tricuspid E velocity in HA-1M, with a further decrease in the HHA group but not the LHA group in HA-1Y. In contrast, the tricuspid A velocity decreased only in HA-1Y, not in HA-1M, for both groups. Notably, there were no significant differences in the dimensional or functional parameters of the RA or RV between the LHA and HHA groups.

**TABLE 5:** Echocardiographic parameters of the right heart at sea level and after 1 month and 1 year of migration to HA.

	SL		HA-1M		HA-1y		ANOVA *p*-value		
	LHA	HHA	LHA	HHA	LHA	HHA	time	group	interaction
RA MD (mm) (95% CI)	35.8 ± 3.3 (35.0–36.7)	35.3 ± 3.5 (34.4–36.2)	33.4 ± 3.3* (32.6–34.3)	32.8 ± 3.7* (31.9–33.7)	32.2 ± 3.0*† (31.4–33.1)	32.2 ± 3.7* (31.3–33.0)	<0.001	0.422	0.625
RA A (cm^2^) (95% CI)	13.7 ± 1.9 (13.2–14.2)	13.6 ± 1.8 (13.1–14.1)	11.6 ± 1.9* (11.2–12.1)	11.5 ± 2* (11.0–12.0)	10.9 ± 1.7*† (10.4–11.3)	11.3 ± 1.8* (10.8–11.8)	<0.001	0.788	0.165
RV MD (mm) (95% CI)	31.0 ± 4.0 (30.0–32.0)	30.4 ± 4.0 (29.3–31.4)	28.7 ± 3.5* (27.9–29.5)	28.4 ± 3.1* (27.5–29.3)	28.5 ± 3.0* (27.7–29.3)	28.4 ± 3.5* (27.6–29.3)	<0.001	0.532	0.716
RVOTD (mm) (95% CI)	27.5 ± 3.8 (26.6–28.4)	27.8 ± 3.7 (26.8–28.7)	26.2 ± 3.3* (25.4–27.0)	26.5 ± 3.2* (25.7–27.4)	26.2 ± 3.0* (25.5–27.0)	26.6 ± 2.8* (25.8–27.3)	<0.001	0.540	0.969
RV FWT (mm) (95% CI)	3.27 ± 0.55 (3.12–3.41)	3.32 ± 0.64 (3.16–3.48)	3.34 ± 0.50 (3.22–3.46)	3.35 ± 0.46 (3.23–3.48)	3.26 ± 0.41 (3.16–3.36)	3.25 ± 0.39 (3.14–3.35)	0.231	0.778	0.809
RV EDA (cm^2^) (95% CI)	20.3 ± 4.0 (19.3–21.2)	19.7 ± 3.8 (18.6–20.7)	18.2 ± 3.2* (17.4–19.0)	18.2 ± 3.2* (17.3–19.0)	18.1 ± 3.3* (17.3–18.8)	18.4 ± 3.0* (17.5–19.2)	<0.001	0.816	0.334
RV ESA (cm^2^) (95% CI)	12.0 ± 3.0 (11.3–12.8)	11.5 ± 2.8 (10.7–12.3)	11.0 ± 2.4* (10.4–11.6)	10.7 ± 2.2 (10.1–11.4)	11.3 ± 2.5 (10.7–11.9)	11.5 ± 2.1† (10.9–12.1)	0.001	0.616	0.250
RV FAC (%) (95% CI)	41.1 ± 5.8 (39.8–42.4)	42.0 ± 4.9 (40.6–43.4)	39.9 ± 4.1 (38.9–41.0)	40.9 ± 4.7 (39.8–42.1)	37.5 ± 4.8*† (36.4–38.6)	37.4 ± 4.3*† (36.2–38.6)	<0.001	0.366	0.487
TAPSE (mm) (95% CI)	23.1 ± 3.3 (22.3–23.9)	23.4 ± 3.2 (22.5–24.2)	19.5 ± 2.9* (18.8–20.3)	19.6 ± 2.8* (18.9–20.4)	19.6 ± 3.1* (18.8–20.4)	20.4 ± 3.1* (19.6–21.3)	<0.001	0.326	0.505
Tricuspid E (cm/s) (95% CI)	62.2 ± 12.3 (59.3–65.0)	58.9 ± 10.8 (55.8–61.9)	51.1 ± 10.1* (48.6–53.5)	50.1 ± 9.9* (47.5–52.7)	49.2 ± 8.9* (47.1–51.3)	45.6 ± 8.3*† (43.4–47.9)	<0.001	0.067	0.376
Tricuspid A (cm/s) (95% CI)	31.2 ± 7.8 (29.4–33.0)	30.7 ± 6.9 (28.8–32.7)	29.8 ± 7.2 (28.1–31.5)	29.6 ± 6.9 (27.7–31.5)	28.2 ± 7.1* (26.6–29.8)	27.5 ± 5.8* (25.8–29.2)	0.001	0.604	0.959
Tricuspid E/A ratio (95% CI)	2.1 ± 0.5 (1.9–2.2)	2.0 ± 0.5 (1.9–2.1)	1.8 ± 0.3* (1.7–1.8)	1.7 ± 0.3* (1.6–1.8)	1.8 ± 0.3* (1.7–1.9)	1.7 ± 0.40* (1.6–1.8)	<0.001	0.143	0.779
PA (mm) (95% CI)	18.9 ± 1.5 (18.5–19.2)	19.2 ± 1.6 (18.8–19.6)	19.7 ± 1.5* (19.3–20.1)	20.0 ± 1.7* (19.5–20.4)	20.3 ± 1.4* (19.9–20.7)	21.4 ± 1.9*†‡ (20.9–21.8)	<0.001	0.005	0.047
PA PSV (cm/s) (95% CI)	98.4 ± 14.6 (94.8–102.0)	96.8 ± 14.8 (93.0–100.7)	92.7 ± 18.4* (88.8–96.6)	88.6 ± 12.7* (84.4–92.8)	89.6 ± 12.8* (86.6–92.6)	85.0 ± 11.4* (81.8–88.1)	<0.001	0.104	.491
IVC (mm) (95% CI)	20.0 ± 3.8 (19.1–20.9)	20.2 ± 3.6 (19.2–21.1)	16.4 ± 4.1* (15.4–17.4)	16.2 ± 3.9* (15.2–17.3)	14.0 ± 3.4*† (13.1–14.8)	14.1 ± 3.5*† (13.2–15.0)	<0.001	0.943	0.924
RAP (mmHg) (95% CI)	5.77 ± 2.50 (5.15–6.39)	5.28 ± 2.51 (4.62–5.94)	4.89 ± 3.35 (4.12–5.67)	4.65 ± 2.94 (3.82–5.48)	4.69 ± 2.38 (4.02–5.36)	5.53 ± 3.06 (4.81–6.24)	0.097	0.914	0.132
TRV (cm/s) (95% CI)	218.9 ± 20.1 (213.0–224.8)	220.6 ± 28.1 (214.3–227.0)	232.0 ± 20.6* (226.3–237.6)	246.7 ± 25.3*‡ (240.7–252.7)	248.1 ± 28.1*† (240.0–256.2)	271.0 ± 37.8*†‡ (262.4–279.7)	<0.001	0.001	0.001
TRPG (mmHg) (95% CI)	19.3 ± 3.4 (18.3–20.4)	19.8 ± 5.1 (18.7–20.9)	21.7 ± 3.9* (20.6–22.8)	24.2 ± 5.0*‡ (23.1–25.4)	24.9 ± 5.7*† (23.1–26.7)	29.9 ± 8.8*†‡ (28.0–31.9)	<0.001	0.001	0.001
PASP (mmHg) (95% CI)	25.1 ± 4.6 (23.9–26.3)	25.1 ± 5.5 (23.7–26.4)	26.6 ± 5.4 (25.2–28.0)	28.9 ± 6.2*‡ (27.4–30.4)	29.6 ± 6.5*† (27.6–31.6)	35.5 ± 9.7*†‡ (33.3–37.6)	<0.001	0.003	<0.001

Data are expressed as mean ± SD.

CI, confidence interval; RA MD, right atrium minor-axis dimension; RA A, right atrial area; RVMD, right ventricular mid-cavity dimension; ROVTD, right ventricular outflow tract diameter; RV FWT, right ventricular free wall thickness; RV EDA, right ventricular end-diastolic area; RV ESA, right ventricular end-systolic area; RV FAC, right ventricular fraction area change; TAPSE, tricuspid annular plane systolic excursion; PA, pulmonary artery; PA PSV, pulmonary artery peak systolic velocity; IVC, inferior vena cava; RAP, right atrial pressure; TRV, tricuspid regurgitation velocity; TRPG, tricuspid regurgitation pressure gradient; PASP, pulmonary artery systolic pressure.

*p < 0.05 vs. SL; †*p* < 0.05 vs. 1 month; ‡*p* < 0.05 LHA, vs. HHA.

Pulmonary artery diameter widened markedly in HA-1M in both groups. The HHA group experienced further widening in HA-1Y, while the LHA group did not. Notably, the pulmonary artery diameters in the HHA group were significantly wider than those in the LHA group in HA-1Y. Both groups exhibited a decrease in pulmonary artery peak systolic velocity in HA-1M, remaining unchanged in HA-1Y with no significant difference between groups. The diameter of the IVC significantly decreased in HA-1M, with a further decrease in HA-1Y for both groups. However, the right atrial pressure (RAP) estimated by the diameter and variation of the IVC remained unchanged with altitude and time. Both groups showed a progressive increase in TRV, TRPG, and PASP over time. In both HA-1M and HA-1Y, the HHA group displayed significantly higher TRV, TRPG, and PASP compared to the LHA group. Abnormalities of HR, LV eject fraction, RV fraction area change, PASP are presented in Table 6.

**TABLE 6:** Abnormalities of HR, EF, RV FAC, PASP after 1 month and 1 year of migration to HA.

	Abnormality threshold	HA-1M			HA-1y		
		LHA	HHA	Total	LHA	HHA	Total
HR	>100 beats/min	4 (3.3%)	2 (1.6%)	6 (4.9)	0 (0%)	0 (0%)	0 (0%)
EF	<52%	0 (0%)	0 (0%)	0 (0%)	0 (0%)	0 (0%)	0 (0%)
RV FAC	<35%	6 (4.9%)	5 (4.1%)	11 (9.0%)	17 (13.9%)	20 (16.4%)	37 (30.3%)
PASP	>30–50 mmHg	11 (9.0%)	21 (17.2%)	22 (18.0%)	29 (23.8%)	33 (27.0%)	62 (50.8%)
	>50–70 mmHg	1 (0.8%)	3 (2.5%)	4 (3.3%)	5 (4.1%)	8 (6.6%)	13 (10.7%)
	>70 mmHg	0 (0%)	0 (0%)	0 (0%)	0 (0%)	5 (4.1%)	5 (4.1%)

Data are expressed as number (percentage).

HR, heart rate; EF, eject fraction; RV FAC, right ventricular fraction area change; TAPSE, tricuspid annular plane systolic excursion; PASP, pulmonary artery systolic pressure.

## 4 Discussion

This investigation prospectively examined the impact of 1 month and 1 year of high-altitude exposure on cardiac structure and function in healthy young males across varying altitudes. Our key findings include: (i) Both the left and right atria and ventricle exhibited reductions in size, while the PA dilated, likely due to a combination of decreased blood volume and intensified hypoxic pulmonary vasoconstriction. Interestingly, chamber size reductions occurred at a faster rate with increasing altitude. (ii) Compromised bi-ventricular diastolic function emerged within 1 month of HA exposure, followed by deteriorated bi-ventricular systolic function after 1 year. Notably, these functional changes were independent of altitude. (iii) PASP progressively increased with both the duration and altitude of HA exposure. (iv) Echocardiographic markers of blood volume, including diminished IVC diameter and reduced bi-ventricular inflow velocities, indicated that blood volume decline over time, while no significant altitude-related differences were observed.

### 4.1 Cardiac remodeling

Over the years, studies examining the RV area in healthy lowlanders upon initial HA exposure have yielded inconsistent results, reporting slight increases (De Boeck et al., 2018), no changes (Maufrais et al., 2017; Stembridge et al., 1985a), or decreases after prolonged acclimatization (after more than 5 years) (Chen et al., 2022). Additionally, RV dimensions vary across populations at different altitudes: lower in Himalayan Sherpa (5,050 m) (Stembridge et al., 1985a), higher in Peruvian Andeans at La Rinconada (5,100 m) (Doutreleau et al., 2022), and similar to lowlanders in Tibetans (4,300 m) (Chen et al., 2022) and Peruvian Andeans at Puno (3,800 m) (Doutreleau et al., 2022). These discrepancies potentially arise from differences in age, ethnicity, and exposure time among cross-sectional study participants. Our longitudinal study revealed decreases in RV dimensional parameters 1 month and 1 year after HA exposure in healthy lowlanders, mirroring changes in the other three chambers. While LV and LA responses aligned with previous reports, the RA response differed unexpectedly, exhibiting a decrease unlike observations in earlier studies (Chen et al., 2022; Doutreleau et al., 2022).

Previous studies (Alexander and Grover, 1983; Stembridge et al., 1985b; Stembridge et al., 2019) have attributed the reduction in left heart size observed in lowlanders upon HA exposure to 2 mechanisms: decreased blood volume and increased hypoxic pulmonary vasoconstriction. However, Richalet et al. (2024) challenged the hypovolemia hypothesis, arguing that increased red blood cell volume maintains blood volume despite decreased plasma volume. While this is true, it is important to consider the temporal difference: red blood cell increases occur several weeks after HA exposure (Siebenmann et al., 1985a; Siebenmann et al., 1985b; Sawka et al., 2000), leaving a period where the plasma volume decrease remains uncompensated. Several studies have documented a reduction in plasma volume in lowlanders to varying degrees during the first 3 weeks of high-altitude exposure (Alexander et al., 1967; Grover et al., 1998; Robach et al., 1985). This decrease serves as a physiological mechanism to elevate the hemoglobin concentration and arterial oxygen content (Siebenmann et al., 1985a). Additionally, there is extensive evidence demonstrating a concomitant reduction in the total blood volume of lowlanders as plasma volume declines, at least during the initial 2 weeks of HA exposure (Jain et al., 1980; Poulsen et al., 1998; Robach et al., 2002). Our study supports this notion, as echocardiographic indicators of blood volume (inferior vena cava diameter, tricuspid and mitral E- and A-wave velocities significantly decreased after 1 month and 1 year of HA exposure, suggesting a decline during the first year of acclimatization. Furthermore, the observed right heart reduction cannot be solely explained by hypoxic pulmonary vasoconstriction, which typically leads to RV dilation due to elevated pulmonary pressure (Motley et al., 1947; Netzer et al., 2017). Although our study revealed increased PASP and PA dilation, suggesting that this mechanism in play, it cannot fully explain the right heart size decrease. It is highly conceivable that the secondary decrease in RV output, caused by increased PASP, resulted in a reduced left ventricular volume and output through serial interaction (Belenkie et al., 2001). Therefore, we propose that both decreased blood volume and hypoxic pulmonary vasoconstriction contribute to the reduction in size observed in all 4 heart chambers. These physiological mechanisms generally serve as adaptive responses to high-altitude exposure, helping to optimize arterial oxygen content and gas exchange (Williams et al., 2022). However, excessive or prolonged decreases in blood volume and hypoxic pulmonary vasoconstriction can lead to increased blood viscosity, pulmonary hypertension, and heart failure. In such cases, maintaining proper hydration, providing supplemental oxygen, and in some instances, administering medication are necessary (Gatterer et al., 2024).

The observed reductions in LV mass and LV mass index in our study are primarily attributed to decreased LV volume, as evidenced by unchanged wall thickness. Similarly, the lack of change in RV free wall thickness suggests potentially minimal myocardial remodeling following 1 year of hypoxic exposure at high altitude.

### 4.2 Cardiac function

Left and right ventricular systolic function have been shown to be preserved during short-term high-altitude exposure, regardless of altitude. Similarly, native highlanders exhibit this pattern of preserved LV systolic function (Maufrais et al., 2017; Holloway et al., 2011; Stembridge et al., 1985a; Huez et al., 2005). However, reports on RV systolic function in long-term HA residents are mixed, with findings of both decreased and preserved function compared to those in lowlanders (Stembridge et al., 1985a; Doutreleau et al., 2022; Huez et al., 2009; Dedobbeleer et al., 2015). Likewise, chronic mountain sickness patients reportedly have enlarged RVs and impaired RV systolic function (measured by echocardiography), but exercise testing revealed comparable RV contractile reserves to those of healthy highlanders (Pratali et al., 2013). This suggests that echocardiographic parameters may not always accurately reflect true RV systolic impairment. In our study, both the LV ejection fraction and RV fractional area change decreased, with an earlier decline in the TAPSE. While cardiac output was maintained by increased heart rate despite a decrease in SV, SV further dropped in the lower altitude group after 1 year. The early decrease in TAPSE could be secondary to the aforementioned reductions in RV diameter and volume, likely driven by decreased blood volume. Similarly, the decline in the bicuspid and tricuspid E/A ratio may result from diminished blood volume affecting both E-wave velocities. Hence, the presented parameters might not have accurately assessed LV diastolic function in our study. Future investigations should employ blood volume-independent parameters to provide a more reliable assessment of LV diastolic function.

### 4.3 Impact of different altitudes

Consequently, the lower air and arterial oxygen content at higher altitudes likely induces more severe hypoxic pulmonary vasoconstriction, possibly explaining a key finding of our study: a higher degree of PA dilation and PASP increase with increasing altitude. This finding aligns with previous reports (Doutreleau et al., 2022; Netzer et al., 2017; Sylvester et al., 2012; Sime et al., 1963). Besides, while the dimensional parameters of the LV, LA, and RA remained comparable between altitude groups, these parameters decreased more rapidly in higher altitude groups. However, the timing and degree of bi-ventricular function decline did not differ across altitudes.

### 4.4 Limitations

This investigation is subject to several limitations. First, the study population was restricted to healthy young males of Chinese ethnicity. This limits the generalizability of our findings to other populations, particularly females. Future research should investigate potential differences in cardiac remodeling and functional changes in females. Second, we were restricted from using traditional echocardiographic parameters due to equipment limitations. As we were unable to use Tissue Doppler Imaging, we could not make a complete assessment of LV diastolic function and RV systolic function. Utilizing more advanced equipment in future studies would allow for longer-term tracking and potentially reveal additional insights. Third, we were failed to include a control cohort staying at low altitude. So, the impact of potential confounding factors such as physical activity, diet, and genetic predispositions cannot be rule out. Incorporating a sea-level control group in future research could provide a more solid basis for comparing and interpreting the observed changes.

## 5 Conclusion

Our findings demonstrated a reduction in the size of cardiac chambers and a mild decline in bi-ventricular function following high-altitude exposure. These changes are likely attributable to a combination of decreased blood volume and increased hypoxic pulmonary vasoconstriction. Notably, higher altitudes were associated with increased PASP and a faster rate of chamber size reduction. However, all observed parameters of cardiac structure and function remained within the normal range, suggesting no clinically significant impairment during a year-long HA stay.

## Data Availability

The raw data supporting the conclusions of this article will be made available by the authors, without undue reservation.
