# Supplementary material for: The impact of high-altitude migration on cardiac structure and function: a 1-year prospective study
Source: Front Physiol. 2024 Aug 30;15:1459031. doi: 10.3389/fphys.2024.1459031 (PMC11392884; doi:10.3389/fphys.2024.1459031)
Supplement: Supplementary file 1 [file DataSheet1.docx]

**2 Materials and methods**

## 2.4 Echocardiography

The measurement protocol of each echocardiographic parameters are as follows:

| Parameter | Technique | Method |
| --- | --- | --- |
| Left atrial anteroposterior diameter | 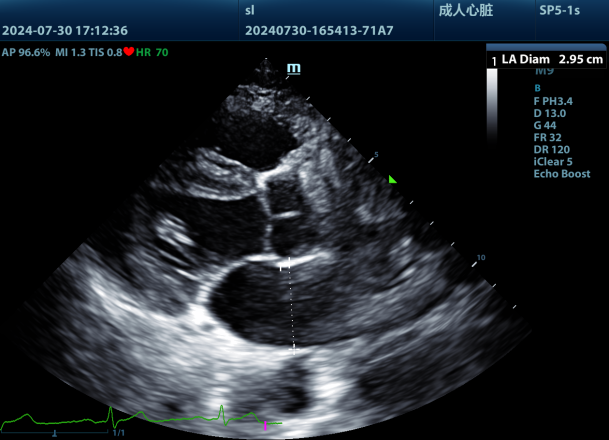 | Measured in parasternal long-axis view, at end-systole, at the level of the aortic sinuses by using the leading-edge to leadingedge convention |
| Left atrial volume | 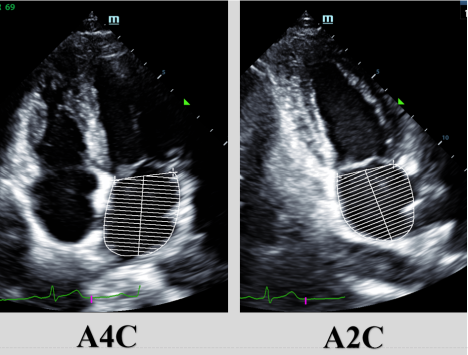 | Measured in four-chamber  apical view and two-chamber  apical view, at end-systole. At the mitral valve level, the contour is closed by connecting the two opposite sections of the mitral annulus with a straight line. |
| Left atrial volume index |  | Calculated by dividing the left atrial volume by the body surface area. |
| Interventricular septal thickness (IVST); left ventricular posterior wall thickness (PWT); left ventricular end-diastolic diameter (EDD) | 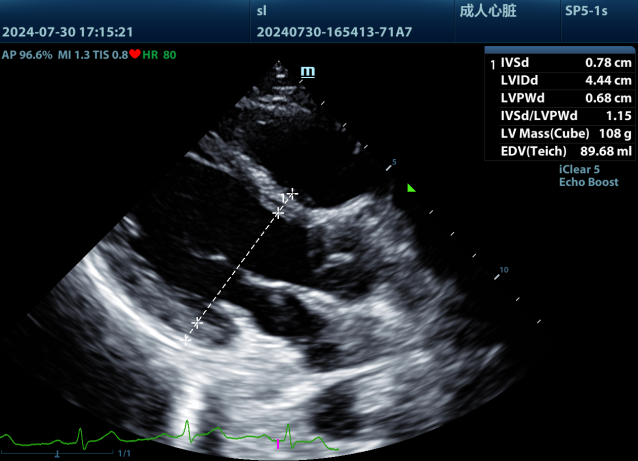 | Measured in parasternal long-axis view, at end-diastole, at the level of the mitral valve leaflet tips, perpendicular to the LV  long axis. |
| Left ventricular (LV) end-diastolic volume (EDV); LV end-systolic volume (ESV); stroke volume (SV); eject fraction (EF) | 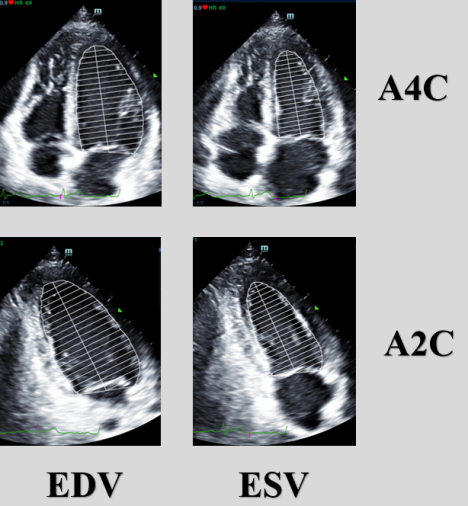 | LV volumes were measured in the apical four- and two-chamber views, at the mitral valve level, the contour is closed by connecting the two opposite sections of the mitral ring with a straight line.  SV= EDV-ESV  EF (%)= 100*SV/EDV |
| Cardiac output |  | Calculated by multiplying stroke volume by heart rate. |
| LV mass |  | Calculated using the linear method:  LV mass= 0.8*1:04*[(IVST+  EDD+PWT)^3^-EDD^3^]+0.6g |
| LV mass index |  | Calculated by dividing LV mass by the body surface area |
| Bicuspid E; Bicuspid A; Bicuspid E/A ratio | 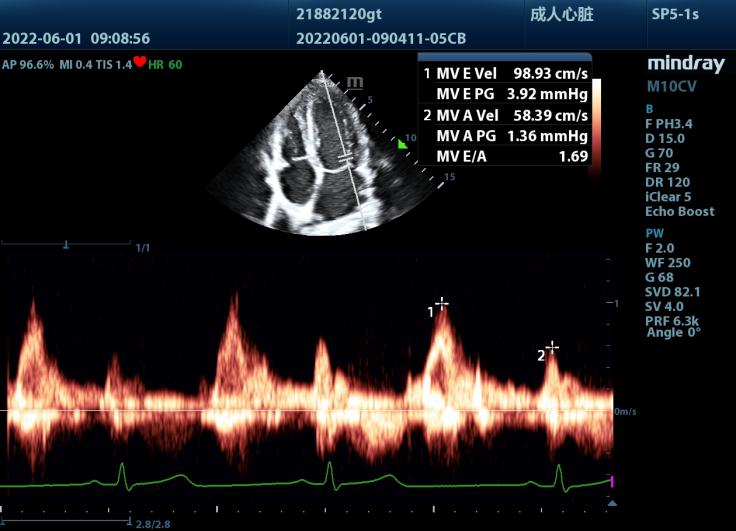 | Measured in four-chamber  apical view, using pulsed Doppler to obtain mitral early (E) and atrial (A) velocity. |
| Right atrium (RA) minor-axis dimension; RA area | 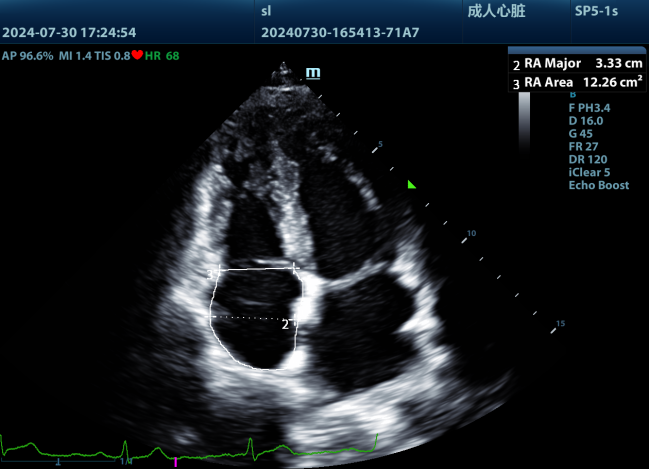 | Measured in the apical  four-chamber view, at end-systole. RA minor-axis dimension was measured at the midatrial level defined by half of RA long axis. RA area was measured by tracing the RA blood-tissue interface. |
| Right ventricular (RV) mid-cavity dimension | 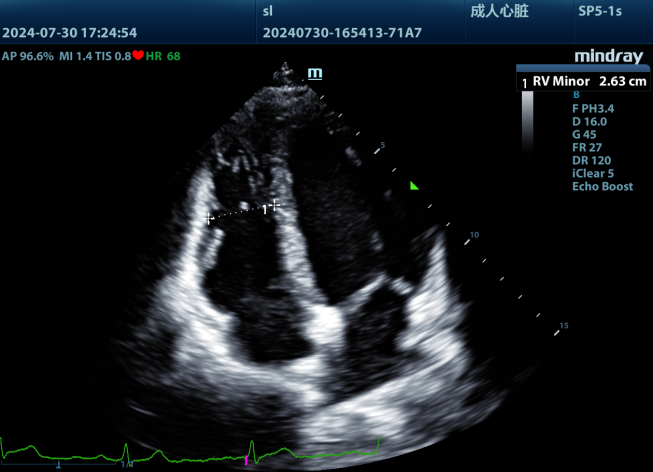 | Measured in the apical  four-chamber view, at end-diastole, at the level of papillary muscles |
| RV outflow tract diameter | 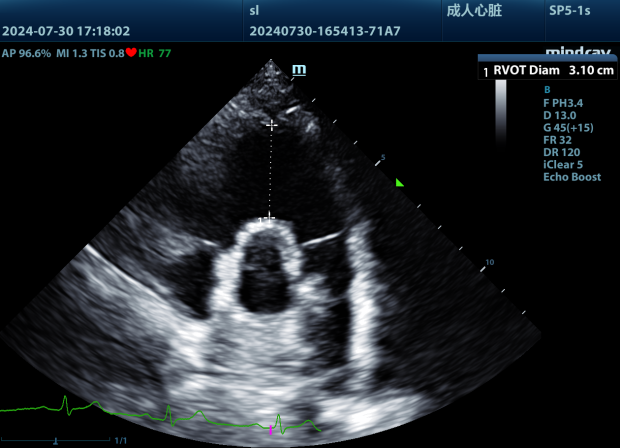 | Measured in parasternal short-axis, at end-diastole, from the anterior RV wall to the aortic valve. |
| RV free wall thickness | 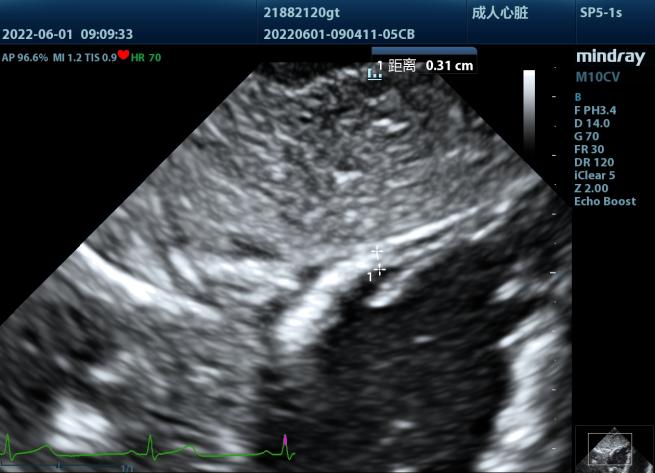 | Measured in subcostal four-chamber view, zoom of region, at end-diastole, below the tricuspid annulus at a distance approximating the  length of anterior tricuspid leaflet. |
| RV end-diastolic area (EDA); RV ventricular end-systolic area (ESA); RV fraction area change (FAC) | 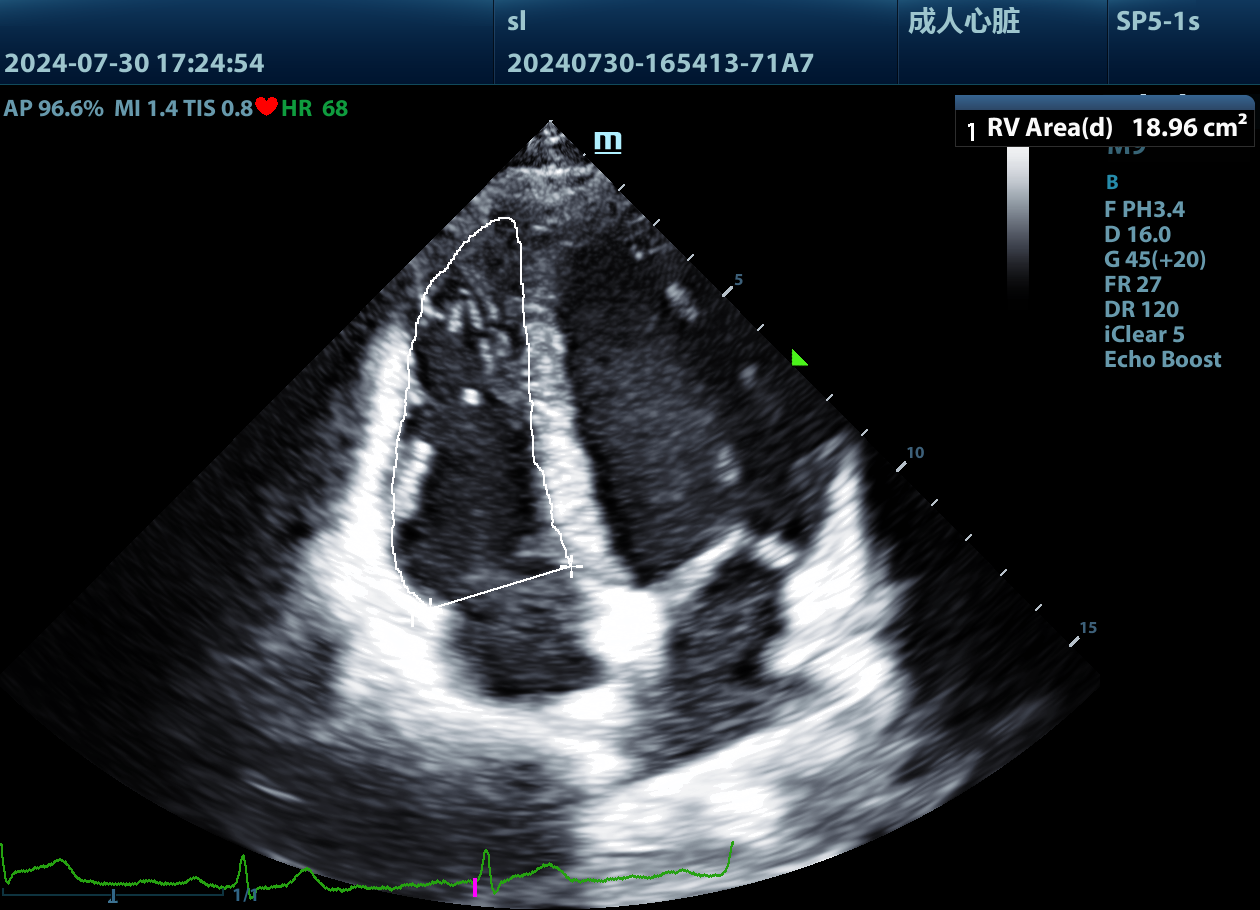  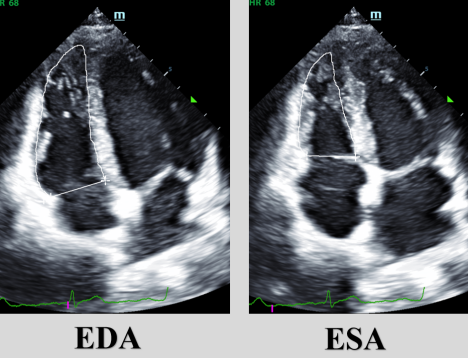 | Manual tracing of RV endocardial border from the lateral tricuspid annulus along the free wall to the apex and back to medial tricuspid annulus in RV-focused four-chamber view, along the interventricular septum at end-diastole and at end-systole. RV FAC (%)=100*(EDA-ESA)/EDA |
| Tricuspid annular plane systolic excursion | 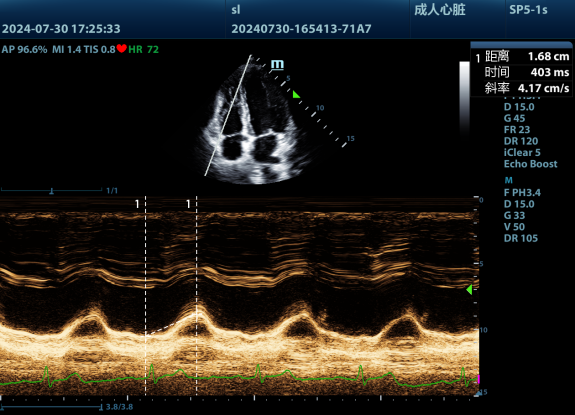 | Tricuspid annular longitudinal  excursion by M-mode, measured between  end-diastole and peak systole. |
| Tricuspid E; Tricuspid A; Tricuspid E/A ratio | 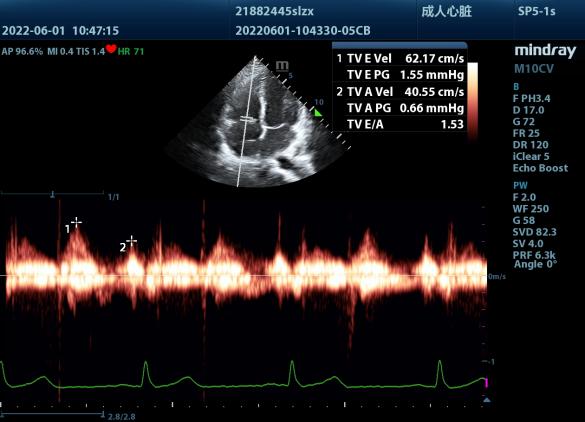 | Measured in four-chamber  apical view, using pulsed Doppler to obtain tricuspid early (E) and atrial (A) velocity. |
| Pulmonary artery | 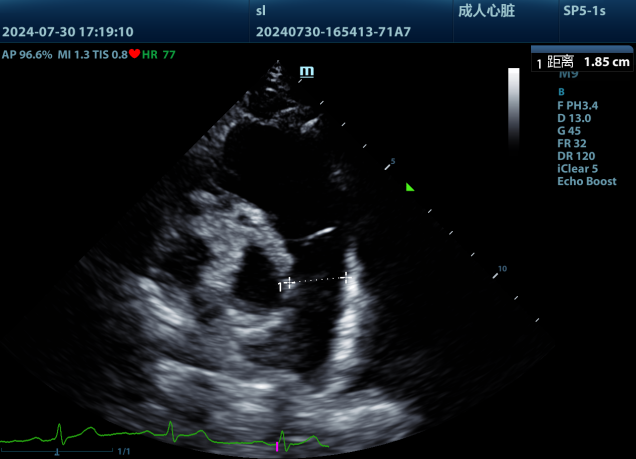 | Measured in parasternal short-axis, at end-diastole, at 1 cm below the pulmonary valve annulus. |
| Pulmonary artery peak systolic velocity | 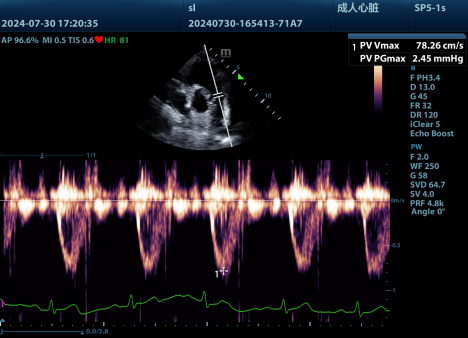 | Measured in parasternal short-axis, using pulsed Doppler to obtain pulmonary artery peak systolic velocity. |
| Inferior vena cava (IVC) | 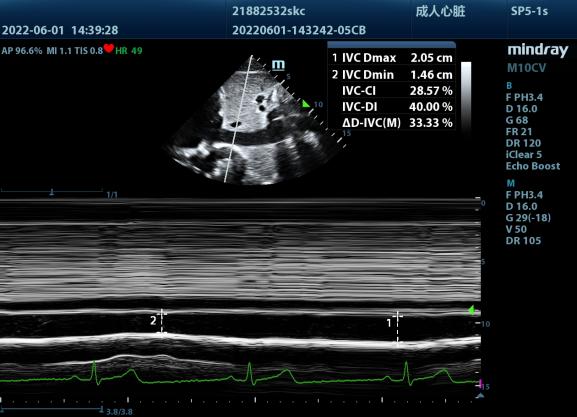 | The diameter of IVC was measured at end-expiration and just proximal to the junction of the hepatic veins that lie approximately 0.5 to 3.0 cm proximal to the ostium of the RA, in subcostal IVC long axis view. The presence of inspiratory collapse of inferior vena cava was also measured in this view. |
| RA pressure |  | Estimated by IVC diameter and the presence of inspiratory collapse. IVC diameter ≤2.1 cm that collapses >50% with a sniff suggests a RA pressure of 3 mm Hg, whereas an IVC diameter >2.1 cm that collapses <50% with a sniff suggests a RA pressure of 15 mm Hg. RA pressure was estimated to be 8 mm Hg in an indeterminate case. |
| Tricuspid regurgitation velocity; tricuspid regurgitation pressure gradient | 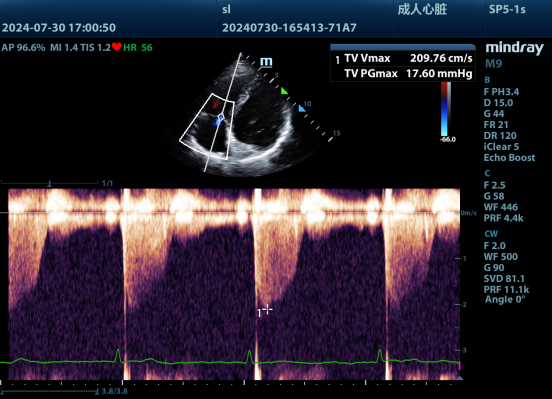 | Measured in four-chamber  apical view, using CW Doppler to obtain highest tricuspid regurgitation velocity and pressure gradient. |
| Pulmonary artery systolic pressure |  | Pulmonary artery systolic pressure =RA pressure+tricuspid regurgitation pressure gradient |
